# The influence of lentivirus-mediated CXCR4 RNA interference on hepatic metastasis of colorectal cancer

**DOI:** 10.3892/ijo.2014.2348

**Published:** 2014-03-19

**Authors:** TIAN-BAO WANG, BAO-GUANG HU, DA-WEI LIU, HAN-PING SHI, WEN-GUANG DONG

**Affiliations:** 1 Departments of Surgery, The First Affiliated Hospital, Sun Yat-sen University, Guangzhou, Guangdong 510080;; 2 Pathology, The First Affiliated Hospital, Sun Yat-sen University, Guangzhou, Guangdong 510080;; 3 Department of Surgery, Prince of Wales Hospital, The Chinese University of Hong Kong, Shatin, Hong Kong, P.R. China

**Keywords:** CXCR4, RNA interference, lentivirus, SW480 cell, hepatic metastasis

## Abstract

The aim of this study was to construct a lentiviral vector of CXCR4-siRNA (Lenti-CXCR4-siRNA) and investigate whether the vector can inhibit the growth, migration, invasion and hepatic metastasis of colorectal cancer (CRC). RT-PCR and western blotting were employed to identify the ideal RNA interference sequence. Lenti-CXCR4-siRNA was constructed and transfected into the SW480 cell line. We used RT-PCR and western blotting to measure the expression of CXCR4 RNA and protein, respectively; the MTS assay to assess the proliferation of SW480 cells; transwell chambers to estimate the inhibitory effect on migration and invasion; and the Balb/c nude mouse model of CRC to examine the inhibition of hepatic metastasis. The relative expression of the CXCR4 gene and protein was 5.4 and 18.95%, respectively, in the siCXCR4 group. The genes in the expression plasmid pLenti-CXCR4-siRNA were in the correct order. In the SW480, nonsense control (NC) and the Lenti-CXCR4-siRNA groups CXCR4 RNA levels were, respectively, 0.54±0.06, 1.00±0.03 and 0.11±0.04 (P=0.0001); CXCR4 protein levels were 0.60±0.03, 0.72±0.03 and 0.18±0.02 (P=0.0001); the OD value was 1.38±0.04 (P=0.0050), 1.28±0.05 (P=0.0256) and 0.92±0.06; SW480 cell number in migration test was 32±6.85, 32.63±1.69 and 0.75±0.71 (P=0.0000); SW480 cell number in the invasion test was 29.13±10.3, 30.38±6.09 and 0.63±0.74 (P=0.0000); hepatic metastasis number was 7.10±3.98 (P=0.034), 7.50±4.09 (P=0.019) and (3.50±2.51); hepatic metastasis mean weight (in g) was 2.25±2.51 (P=0.000), 2.11±2.38 (P=0.000) and 1.45±2.07. Lenti-CXCR4-siRNA constructs were correctly constructed and effectively inhibit the expression of CXCR4 RNA and protein, reducing the proliferation, migration, invasion capacity of SW480 cells and hepatic metastasis of CRC.

## Introduction

Colorectal cancer (CRC) is a common malignant disease. Five-year survival rate and cancer-specific 5-year survival rate of patients with CRC (independent of stage and cause of death) are 56 and 64%, respectively. Overall survival is 96% in stage 1, 92% in stage 2, 58% in stage 3, and 0% for patients with metastatic disease at the time of diagnosis. Cancer-specific survival rates range from 100% for patients in stage 1 to 0% for patients with metastatic disease. Local and distant metastasis are the main risk factors reducing survival of patients with CRC (1).

The process of cancer metastasis includes cancer cell proliferation, local invasion, intravasation and cancer cell survival, extravasation and attachment to secondary organs, and metastatic growth in a new environment (2). CXC chemokine receptor 4 (CXCR4) and its ligand stromal cell-derived factor-1α (SDF-1α, also known as CXCL12) play an important role in cancer growth and dissemination, and CXCR4/SDF-1α is positively correlated with metastasis in many types of cancer including thyroid, lung, ovarian, renal, prostate, breast, pancreatic, gastric and colorectal cancer (3–12).

CXCR4 and SDF-1 have been investigated as growth and metastasis inhibitors of many different cancers. Treatment of anaplastic thyroid carcinoma cells with SDF-1 induces proliferation, which is blocked by the specific CXCR4 antagonist AMD3100 and by CXCR4 RNA interference, and AMD3100 effectively reduces tumor growth in nude mice inoculated with different anaplastic thyroid carcinoma cells (13). Blocking of the SDF-1/CXCR4 interaction with AMD3100 inhibits meta-static tumor growth in a mouse hepatic metastasis model of colon cancer (11). AMD3100 significantly inhibits the invasion ability of SW480 cells and markedly reduces the expression of VEGF and MMP-9 but not MMP-2 (14). Systemic administration of d-Arg3FC131, a CXCR4 antagonist, inhibits the growth of GH3 somatotrope tumor cell xenografts in immunodeficient nude mice by inducing apoptosis and suppressing the proliferation of tumor cells (15). SW620 cell lines with reduced expression of CXCR3 and/or CXCR4 created using microRNA, CXCR3-, CXCR4-, and CXCR3/ CXCR4 double-knockdowns significantly reduces metastasis to lymph nodes, liver and lungs, and significantly decreases the dissemination of cancer cells to liver and lungs (16). Functional CXCR4 knockdown using lentiviral short hairpin RNA (shRNA) vectors significantly decreases the migration behavior in CRC SW480 and SW620 cell lines, and pharmacologic inhibition of the SDF-1α/CXCR4 interaction by the bicyclam plerixafor at 100 *μ*M significantly abrogates CXCR4-dependent migration and invasion (17). Blocking CXCR4 expression at the mRNA level by a combination of two siRNAs impairs invasion of breast cancer cells in the Matrigel invasion assay and inhibits breast cancer metastasis in an animal model (18). Knockdown of CXCR4 significantly limits the growth of orthotopically transplanted breast cancer cells and prevents primary tumor formation in some mice, and all mice transplanted with CXCR RNA interference (RNAi) cells survived without developing macroscopic metastases (19). CXCR4-miRNA-transfected breast tumor cells show reduced migration and invasion ability *in vitro* and formed fewer lung metastases *in vivo* compared to ctrl-miRNA-transfected cells (20).

To date, the relationship between CXCR4 RNAi and the inhibition of metastasis of CRC to the liver has not been well documented. In this study, a lentivirus vector was successfully constructed to introduce CXCR4 RNAi into CRC xenografts. The results demonstrate the efficiency of the lentivirus system to silence CXCR4 and inhibit the growth of hepatic metastasis from grafted CRC. This approach may have therapeutic potential in CRC.

## Materials and methods

### 

#### Main reagents and instruments

Mouse monoclonal anti-human CXCR4 antibody, GAPDH, RPMI-1640, fetal bovine serum (FBS), penicillin, streptomycin, Polybrene, phosphate-buffered saline (PBS; Hyclone, Logan, UT, USA), Matrigel (BD Biosciences, San Jose, CA, USA), RNA extraction kit, reagents for reverse transcription, western blotting kit (Boster Biological Technology Co., Wuhan, China), human colon cancer cell line (SW480 cells), nude (*nu/nu*) BALB/c mice (Shanghai Cancer Institute, Shanghai, China), Lenti-X Bicistronic Expression System (Clontech, Palo Alto, CA, USA), Lipofectamine 2000, Lipofectamine™ RNAiMAX transfection reagents (Invitrogen Corp., Carlsbad, CA, USA), Opti-MEM medium (Gibco-BRL, Gaithersburg, MD, USA), CellTiter96AQ cell proliferation detection kit (Promega, Madison, WI, USA), Transwell plates (BD), SYBR Green PCR Master Mix (Toyobo Biologics, Osaka, Japan), and thermal cycler (ABI PRISM^®^ 7700 Sequence Detection System, Applied Biosystems, Foster City, CA, USA) were used in the present study. This study was approved by the ethics board at the First Affiliated Hospital of Sun Yat-sen University.

#### Synthesis of siRNA

Three pairs of siRNAs targeting CXCR4 [the human *CXCR4* gene (NM-004363.2)] were synthesized by Qiagen: sequence A (siCXCR4-A): 5’-UAAAAUCUUCCUGC CCAC CdTdT-3’ (sense) and 3’-dTdTAUUUUAGAAGGACGG GUGG-5’ (antisense); sequence B (siCXCR4-B): 5’-CAAGGAA GCUGUUGGCUGAdTdT-3’ (sense) and 3’-dTdTGUUCCUU CGACAACCGACU-5’ (antisense); sequence C (siCXCR4-C): 5’-CUGUCCUGCUAUUGCAUUAdTdT-3’ (sense) and 3’-dTd TGACAGGACGACGAUAACGUAAU-5’ (antisense). In addition, negative control siCXCR4 was synthesized by Guangzhou RiboBio Co. (Guangzhou, China) for monitoring the influence of exogenous genes.

#### Culture of SW480 cells and siRNA transfection

SW480 cells were routinely cultured and seeded on 6-well plates at a density of 5×10^4^ cells/well. Then, nonsense control siCXCR4(NCsiCXCR4), siCXCR4-A, siCXCR4-B and siCXCR4-C at different concentrations (25, 50 and 100 nM) were separately added to each well. When 40% confluent, SW480 cells were transfected. The complete medium was removed and cells were washed in PBS twice and maintained in 1 ml of high glucose DMEM containing 20% FBS. The solutions in tube A [i.e., siRNA solution (20 *μ*M) prepared with RNAase-free deionized water, diluted in 500 *μ*l of Opti-MEM, and kept at room temperature] and tube B (5 *μ*l of Lipofectamine 2000 mixed in 500 *μ*l of Opti-MEM and kept at room temperature for ≤5 min) were mixed, kept at room temperature for 20 min, added to each well, and incubated 4-6 h. Then, the medium was removed, the cells were washed in PBS twice, and 2 ml of complete medium was added. At 24 h after transfection, the medium was removed, 1 ml of TRIzol reagent was added, and quantitative PCR was performed to detect mRNA levels.

#### RT-PCR

Total RNA was extracted using a kit according to the manufacturer’s instructions and the quality of RNA determined. In brief, a solution of 1.0 *μ*g of RNA in distilled water (dH_2_O) was diluted to a final volume of 12 *μ*l with dH_2_O, was heated to and kept at 65°C for 5 min for RNA denaturation, kept on ice to avoid RNA renaturation, and incubated with 0.5 *μ*l of Oligo(dT) (Promega) random primer (Promega), 2.0 *μ*l of 10 mM dNTP, 0.5 *μ*l of RNase inhibitor, 4.0 *μ*l of 5X buffer and 0.5 *μ*l of M-MLV at 30°C for 10 min, 42°C for 60 min and 80°C for 10 min. The primers were as follows: 18S rRNA-112 bp (F: CCTGGATACCGCAGCTAGGA; R: GCG GCGCAATACGAATGCCCC). CXCR4-159 bp: (F: ATCAG TCTGGACCGCTACCT; R: GTCATCTGCCTCACTGA CGT). The reaction mixture (including 0.5 *μ*l of cDNA, 0.5 *μ*l of forward primer, 0.5 *μ*l of reverse primer, 10.0 *μ*l of 2X SYBR Green PCR Master Mix, and 4.0 *μ*l of dH_2_O) was kept at 95°C for 5 min, and PCR conditions were as follows: 40 cycles of 95°C for 15 sec, 60°C for 15 sec and 72°C for 32 sec. The melting temperature was 60–95°C. All experiments were performed in triplicate. Cells transfected with 25 nM siCXCR4-C had the lowest expression of CXCR4 and therefore it was used in the following experiment.

### Western blot assays

#### Transfection with siCXCR4-C and NCsiCXCR4

SW480 cells were passaged 24 h prior to transfection. When 30–50% confluent, cells were transfected with 25 nM siCXCR4-C or NCsiCXCR4 in the presence of Lipofectamine™ RNAiMAX in Opti-MEM for 4 h, and then the medium was replaced with RPMI-1640 containing 10% FBS. Protein extraction, SDS-PAGE, and protein transfer were done according to the manufacturer’s instructions. The membrane was washed with Tris-buffered saline with Tween-20 (TBST; 3X, 5 min each time), incubated with 5% non-fat milk at room temperature overnight to block nonspecific binding, washed with TBST (3X, 5 min each time), treated with primary antibody (anti-CXCR4 or anti-GAPDH) at 37°C for 2 h, washed with TBST (3X, 5 min each time), treated with secondary antibody at 37°C for 1 h, washed with TBST (3X) and then with distilled water (3X, a total of 2 min), placed on a plastic board, incubated with chemiluminescence substrate for 5 min, and visualized by an imaging system in the dark.

#### Construction and identification of pLenti-CXCR4-siRNA Splicing of vector GV115 with restriction enzymes

Vector GV115 was digested with *Age*I/*Eco*RI by incubating 3 *μ*l of vector GV115 with 0.5 *μ*l of *Eco*RI, 0.5 *μ*l of *Age*I, 1 *μ*l of 10X D buffer, and 5 *μ*l of deionized water or distilled water at 37°C overnight, and the products were separated by 1% gel electrophoresis.

#### Insertion of siRNA into vector GV115

The following reagents were added to a 0.2-ml EP tube: PCR products (3 *μ*l), recycled vector GV115 (2 *μ*l), 10X ligase buffer (1 *μ*l), T4 DNA ligase (1 *μ*l), and deionized water (3 *μ*l). Ligation was done at 16°C for 2 h. The lentiviral plasmid product was pLenti-CXCR4-siRNA. In the control group, water was added instead of PCR products.

#### Transduction of pLenti-CXCR4-siRNA

On ice, 5 *μ*l of ligation products was added to 50 *μ*l of DH5α competent cells and incubated on ice for 30 min and at 42°C for 90 sec. This solution was rapidly transferred to ice, incubated on ice for 2 min, incubated with 200 *μ*l of LB medium at 37°C for 1 h with mixing at 200 rpm, and transferred to an LB plate containing 100 *μ*g/ml ampicillin (Amp). The plate was incubated at room temperature until the solution was absorbed, and inverted and placed in a 37°C incubator overnight.

#### Identification of pLenti-CXCR4-siRNA

One colony was collected from the plate, transferred to a 3-ml LB tube, and incubated overnight with continuous shaking for plasmid extraction. The sequencing of pLenti-CXCR4-siRNA was performed by the BGI Gene Tech Co., Ltd. (Beijing, China). There was a *Xho*I restriction site in the pLenti-CXCR4-siRNA sequence, and thus *Xho*I was used for identification. Once the sequence was cleaved, these plasmids were either positive or negative for pLenti-CXCR4-siRNA. The plasmid was cut with restriction enzyme *Xho*I at 37°C overnight in the following solution: 3.6 *μ*l of ddH_2_O, 1 *μ*l of 10X H buffer, 1 *μ*l of 10X BSA, 4 *μ*l of plasmid and 0.4 *μ*l of *Xho*I.

#### Packaging of Lenti-CXCR4-siRNA

293T cells in the logarithmic phase (cell confluence: 70–80%) were washed with 5 ml of PBS, treated with 2–3 ml of trypsin until these cells became round, switched to complete medium [90% DMEM (high glucose: 4.5g/l) + Glu Max + 10% FBS + penicillin + streptomycin (100X)] to stop the cutting, and dispersed into a single cell suspension, counted to determine cell density, and seeded at 3×10^5^ cells/ml into flasks (10 ml/flask). At 30 min before transfection, the medium was replaced with complete medium (5 ml/flask). The packaging of lentiviral vector was done according to the instructions provided with the Lenti-X Bicistronic Expression System. In brief, 1.5 *μ*g of packaging plasmid, 0.5 *μ*g of expression plasmid, and 250 *μ*l of serum-free medium in a 1.5-ml sterilized EP tube were incubated at room temperature for 5 min, while 9 *μ*l of Lipofectamine 2000 and 250 *μ*l of serum-free medium in a separate 1.5-ml sterilized EP tube were incubated at room temperature for 5 min. The DNA solution and Lipofectamine were mixed and incubated at room temperature. To each well of a 6-well plate, the following was added with overnight incubation at 37°C: 1 ml of growth medium containing serum and DNA-Lipofectamine mixture and 1 ml of re-suspended 293T cells (1×10^6^ cells/ml). The medium containing DNA-Lipofectamine was removed and replaced by DMEM. At 48–72 h after transfection, the supernatant was collected.

#### Condensation and determination of titer of Lenti-CXCR4-siRNA

NaCl (5 mol/l) was added to the above supernatant to a final concentration of 0.15 mol/l. A solution of 20% (w/v) PEG8000 was added and the final mixture was incubated at 4°C overnight with continuous shaking, then centrifuged at 4°C for 30 min at 10,000 g. The supernatant was collected with a vacuum pump in a biological safety cabinet and the sediment was diluted in an appropriate volume of OptiMEM, covered with Parafilm membrane, and stored at 4°C. The Lenti-CXCR4-siRNA was titered using a kit according to the manufacturer’s instructions.

### Effects of Lenti-CXCR4-siRNA on growth, migration, invasiveness, and liver metastasis of SW480 cells

#### Culture of SW480 cells

SW480 cells were seeded into 20 wells of a 96-well plate at a density of 3–5×10^3^ cells/ml (90 *μ*l/well).

#### Transfection of SW480 cells with Lenti-CXCR4-siRNA

Conventional medium was added to 2 sterilized 1.5-ml EP tubes (45 *μ*l per tube). Then, 5 *μ*l of 1×10^8^ TU/ml Lenti-CXCR4-siRNA was serially diluted to 1×10^7^ TU/ml and 1×10^6^ TU/ml by addition to the first tube followed by gentle vortexing to avoid foaming and subsequent transfer of 5 *μ*l of solution in the first tube to the second tube followed by vortexing. In another tube, 2 *μ*l of Polybrene was diluted to 400 and 10 *μ*l added to each well. The volume of Polybrene per well of 100 *μ*l was diluted 2,000 times to a final concentration of 5 *μ*g/ml. Then, 10 *μ*l of lentivirus solution at three different concentrations (1×10^6^, 1×10^5^ and 1×10^4^ TU, respectively), was added to the corresponding wells. The cell count per well was ∼1×10^4^. Thus, the multiplicity of infection (MOI) per well was 100, 10 and 1, respectively. In the Polybrene well, 10 *μ*l of diluted Polybrene was added. The plates were gently vortexed and placed in an incubator for 8–12 h. The cells were observed and the supernatant was removed. The medium was refreshed. At 3–4 days after transfection, fluorescence was detectable under a microscope.

#### Screening of colonies

SW480 cells were seeded into a 6-well plate and maintained in medium containing penicillin and streptomycin on the first day, transfected when 50–60% confluent on the second day, visualized (i.e., counted and marked) as fluorescent colonies on the plate under a fluorescence microscope on the third day. The marked cell colonies were digested with 0.25% trypsin, transferred to a 24-well plate, and observed again under a fluorescence microscope. Those with fluorescence were selected for passaging.

#### Detection of CXCR4 expression by qRT-PCR and western blot assays

The expression of CXCR4 mRNA and protein was assayed by real-time reverse transcription (qRT)-PCR and western blotting as described above.

#### Detection of cell viability (MTS)

Cells were divided into the SW480 group, negative control (NC) group, and Lenti-CXCR4-siRNA group. Cells were digested with trypsin, adjusted to a density of 1×10^5^ cells/ml, seeded into a 96-well plate (100 *μ*l/well; 1×10^4^ cells/well), allowed to adhere, collected at 24, 48, 72 and 96 h, incubated 4 h with MTS solution (ratio: 1:10), and assessed for cell proliferation by optical density (OD) measurement at 490 nm with a micro-plate reader.

#### Detection of cell migration after Lenti-CXCR4-siRNA transfection by Transwell assay

Cells were grouped as described above. Two days after transfection of 1×10^5^ cells with Lenti-CXCR4-siRNA, the cells were re-suspended in 100 *μ*l of serum-free RPMI-1640, transferred to the upper chamber of a Transwell system with 600 *μ*l of complete medium added to the lower chamber, and incubated at 37°C in an atmosphere containing 5% CO_2_ for 24 and 48 h. The cells in the upper chamber were removed with a swab, fixed in 4% paraformaldehyde for 15 min, and washed in PBS once. The upper chamber was stained with crystal violet for 10 min and washed in PBS once. The cells migrating from the upper chamber were counted under a microscope.

#### Detection of invasiveness of cells transfected with Lenti-CXCR4-siRNA by Transwell assays

Cells were divided into 3 groups as described above. Approximately 40 *μ*l of Matrigel solution (Matrigel kept at 4°C overnight and then mixed with serum-free medium at a ratio of 1:3) was added to a pre-cooled Transwell system and incubated at 37°C for 2 h. After excess solution was removed from the chamber, serum-free medium (100 and 600 *μ*l, respectively) was added to the upper and lower chambers, and the Transwell plate was incubated at 37°C overnight. The procedures described above were performed.

#### Liver metastasis of SW480 cells after Lenti-CXCR4-siRNA transfection in BALB/c nu/nu mice

A total of 30 BALB/c *nu/nu* mice aged 4–6 weeks and weighing 14–23 g were housed in a specific pathogen-free (SPF) environment. These animals were divided into three groups as described above (n=10). Cells in the logarithmic growth phase were digested in 0.25% trypsin to prepare a single cell suspension. The cell viability was confirmed to be ≥95% by trypan blue exclusion staining. The cell density was adjusted to 1×10^7^/ml. The procedures were done under aseptic conditions. Mice were intraperitoneally anesthetized with 1% pentobarbital sodium (35 mg/kg). A midline incision was made in the upper abdomen, and the spleen was exposed and slowly injected with cell suspension (0.2 ml; 2×10^6^ cells). The injection site was compressed with a tampon soaked in 95% alcohol and the wound was closed. These mice were returned to SPF housing conditions and monitored for activity, food intake, and change in body weight. At 4 weeks after surgery, laparotomy and subsequent exploration were done to observe liver metastasis. The number of metastatic sites and hepatic metastasis mean weight (in g) were determined.

#### Statistical analysis

Statistical analysis was done using SPSS version 11.0 for Windows. Comparisons were made by t-test. A value of P<0.05 was considered statistically significant.

## Results

### 

#### RT-PCR

The mRNA expression was the lowest in the sequence C group and ∼5.4% of that in the NCsiCXCR4 group. Thus, cells with sequence C were used in the following experiments and designated the siCXCR4 group.

#### Western blot assays

**Figure 1.**
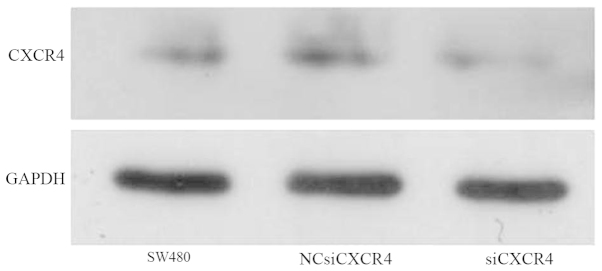
Cells in the siCXCR4 group had the lowest CXCR4 expression.

The SW480, NCsiCXCR4, and siCXCR4 groups had a GAPDH optical density (OD) of 367.67, 3593.84 and 3190.51, respectively and a CXCR4 OD of 820.54, 1106.66 and 604.48, respectively. Thus, the relative expression of CXCR4 was 22.34, 30.80 and 18.95%, respectively. These findings demonstrated that siCXCR4 could effectively inhibit expression of CXCR4 protein (Fig. 1).

#### Cutting of pLenti-CXCR4-siRNA

**Figure 2.**
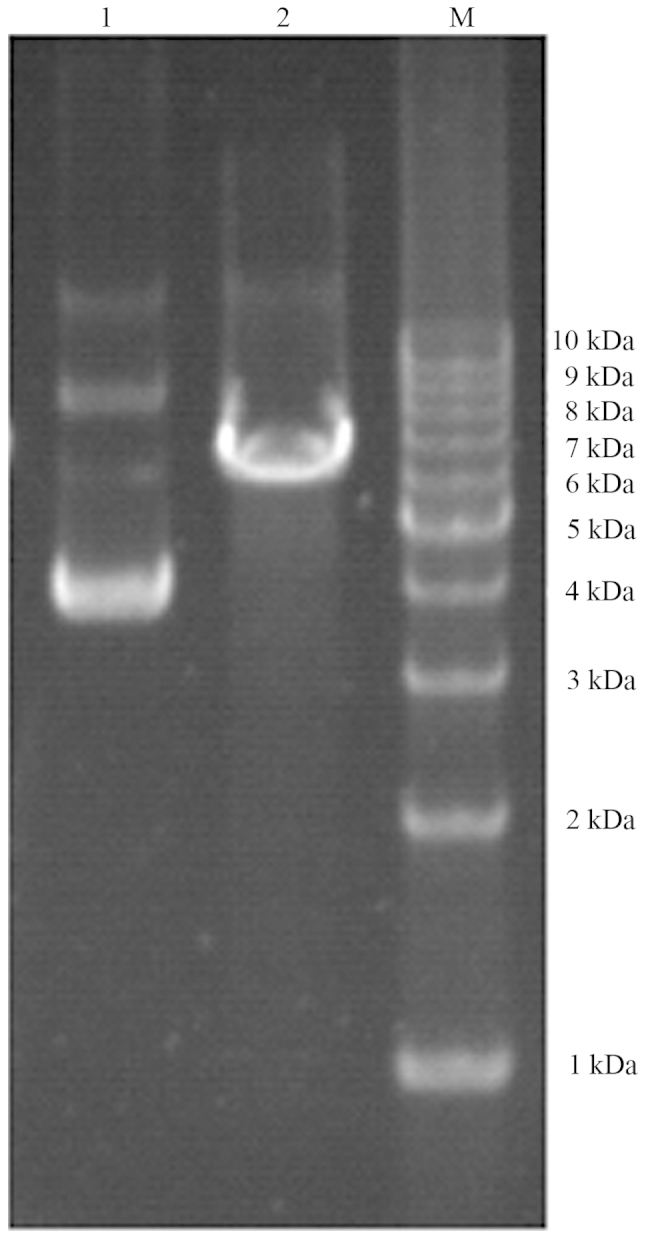
Identification by cutting: M, 1 kb DNA ladder; 1, blank GV115; 2, pLenti-CXCR4-siRNA was cut by *Xho*I restriction enzyme into the linear form.

The vector GV115 (7.5 kb in size) had no *Xho*I restriction site. However, the siRNA sequence had a *Xho*I site that could be cut by *Xho*I. Blank GV115 was circular and therefore migrated more rapidly. Thus, blank GV115 migrated ahead of the cut GV115 (linear sequence), suggesting successful cloning (Fig. 2).

#### Sequencing of pLenti-CXCR4-siRNA

**Figure 3.**
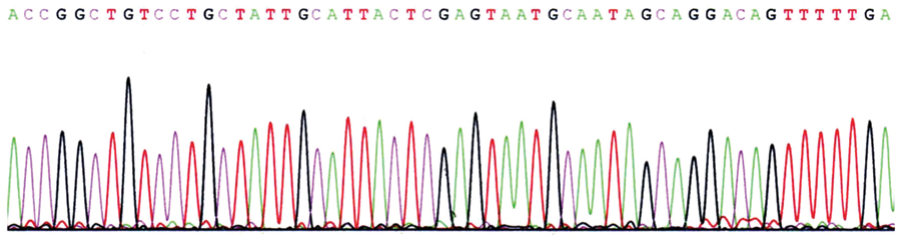
The sequence of pLenti-CXCR4-siRNA was identical to the designed sequence.

All the bases in siRNA were correct. BLAST analysis showed successful cloning and that there was no mutation. Thus, pLenti-CXCR4-siRNA could be used in the experiments that followed (Fig. 3).

#### Transfection of 293T cells with pLenti-CXCR4-siRNA

**Figure 4.**
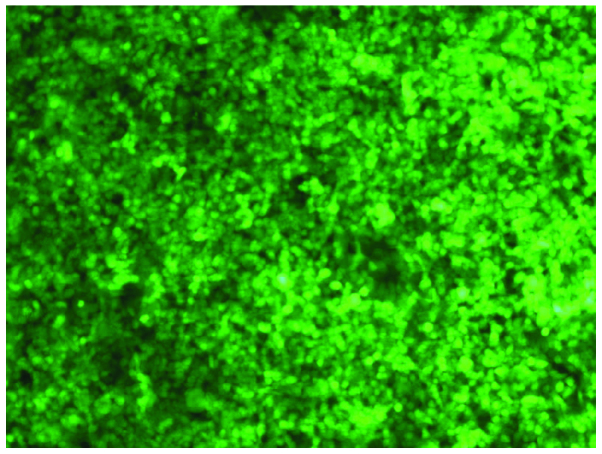
pLenti-CXCR4-siRNA transfected cells show green fluorescence (×100).

All 293T cells transfected with pLenti-CXCR4-siRNA were shown to exhibit green fluorescence under a fluorescence microscope (Fig. 4).

#### Purified Lenti-CXCR4-siRNA (titer 2×10^9^ TU/ml) was used for transfection of SW480 cells

**Figure 5.**
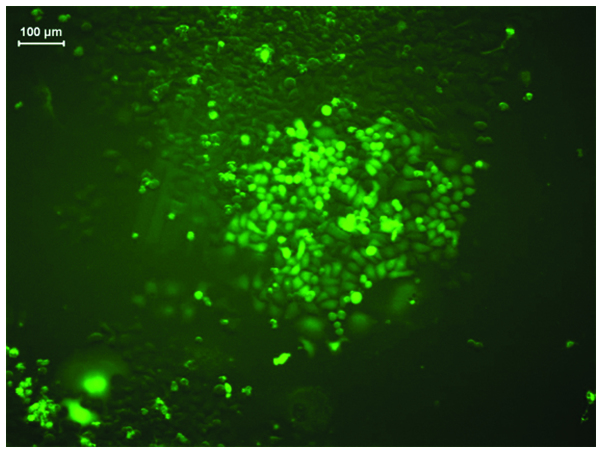
Lenti-CXCR4-siRNA infected SW480 cells show green fluorescence (×100).

All infected cells showed green fluorescence under a fluorescence microscope, suggesting successful transfection (Fig. 5).

#### Detection of CXCR4 mRNA

In the SW480, NC, and Lenti-CXCR4-siRNA groups, the expression of CXCR4 mRNA was 0.54±0.06, 1.00±0.03 and 0.11±0.04, respectively. The expression of CXCR4 mRNA was the lowest in the CXCR4 RNA group and differed markedly from the SW480 and NC groups (P=0.0001).

#### Detection of CXCR4 protein

**Figure 6.**
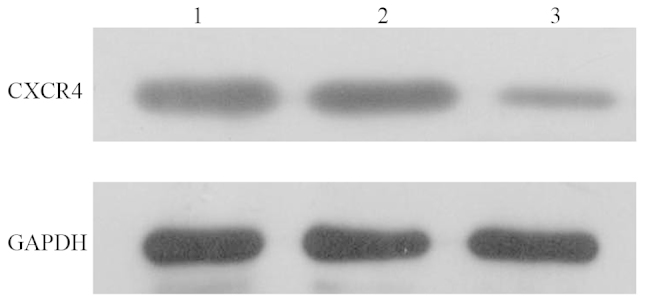
Protein expression of CXCR4: 1, the SW480 group; 2, NC group; 3, Lenti- CXCR4-siRNA group. Cells in the Lenti-CXCR4-siRNA group had the lowest CXCR4 protein expression.

In the SW480, NC, and Lenti-CXCR4-siRNA groups, the expression of CXCR4 protein (in *μ*g/*μ*l) was 4.61, 3.58 and 3.08, respectively. Western blot assays showed that the expression of CXCR4 protein was the lowest in the Lenti-CXCR4-siRNA group. The relative expression of CXCR4 protein was 0.18±0.02, 0.6±0.03 and 0.72±0.03, respectively, in the Lenti-CXCR4-siRNA, SW480, and NC groups (Fig. 6, P=0.0001).

#### Detection of MTS

At 4 days after infection, proliferation (OD of MTS) was markedly lower in the Lenti-CXCR4-siRNA group than in the SW480 group (0.92±0.06 vs 1.38±0.04, P=0.0050) and NC group (0.92±0.06 vs 1.28±0.05, P=0.0256). This suggests that RNA interference (RNAi) significantly inhibits the growth of SW480 cells.

#### Detection of cell migration

**Figure 7.**
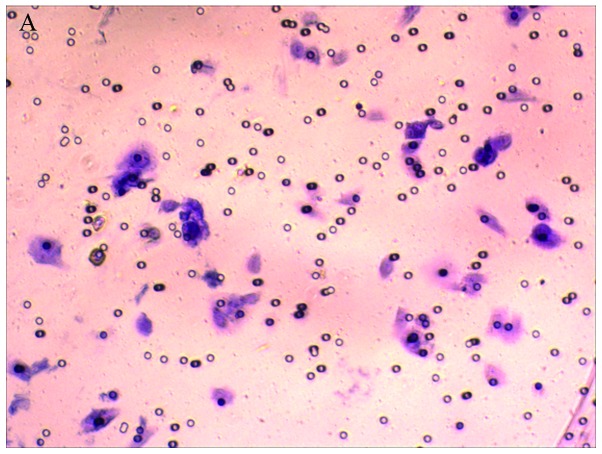

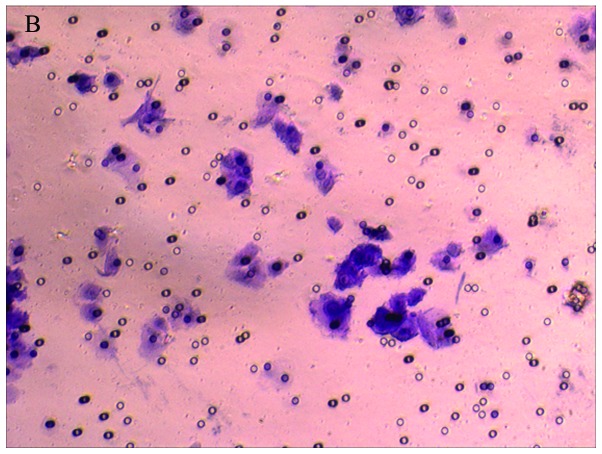

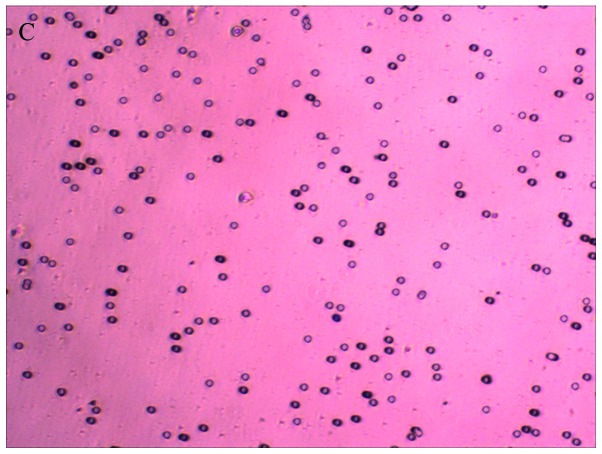
Migration experiment: (A) SW480 group; (B) NC group; (C) Lenti-CXCR4-siRNA group (×100). The number of migrated cells was significantly lower in the Lenti-CXCR4-siRNA group than in the SW480 group and NC group.

The number of migrated cells was significantly lower in the Lenti-CXCR4-siRNA group than in the SW480 group (0.75±0.71 vs 32±6.85, P=0.0000) and NC group (0.75±0.71 vs 32.63±1.69, P=0.0000). This suggests that RNAi significantly suppresses the migration of SW480 cells (Fig. 7).

#### Detection of cell invasiveness

The number of migrated cells was dramatically lower in the Lenti-CXCR4-siRNA group than in the SW480 group (0.63±0.74 vs 29.13±10.30) and NC group (0.63±0.74 vs 30.38±6.09; P=0.0000). This suggests that CXCR4 RNAi markedly inhibits the invasiveness of SW480 cells.

#### Liver metastasis of SW480 cells after Lenti-CXCR4-siRNA transfection

The number of metastatic lesions was significantly lower in the Lenti-CXCR4-siRNA group than in the SW480 group (3.50±2.51 vs 7.10±3.98, P=0.034) and NC group (3.50±2.51 vs 7.50±4.09, P=0.019). The mean weight of metastatic tumors (in g) was dramatically lower in the Lenti-CXCR4-siRNA group than in the SW480 group (1.45±2.07 vs 2.25±2.51, P=0.000) and NC group (1.45±2.07 vs 2.11±2.38, P=0.000). These findings demonstrate that Lenti-CXCR4-siRNA transfection markedly inhibits the liver metastasis of SW480 cells.

## Discussion

As chemoattractant cytokines, chemokines can facilitate cell activation, differentiation, and trafficking. Several organs including lung, lymph nodes, liver, skeletal muscle, brain, kidney, heart, skin, and bone marrow, express CXCL12.

CXCR4 is expressed in most cancer cells, and hypoxia and injury stimulate its production. The binding of CXCL12 to CXCR4 initiates several signaling pathways and promotes chemotaxis, cell proliferation, increase in intracellular calcium concentration and gene transcription (21). CXCR4-positive cancers metastasize to the lymph nodes, liver, and bones in a CXCL12-dependent manner (22). The CXCL12/CXCR4 pathway plays a pivotal role in several aspects of tumor progression including vascularization, metastasis and survival (23,24). In CRC tissues, CXCL12 is significantly downregulated and CXCR4 is significantly upregulated compared to the corresponding normal tissues (25). The frequency of cytoplasmic and nuclear expression of CXCR4 in CRC was shown to be 35.6 and 36.9%, respectively, and nuclear but not cytoplasmic expression of CXCR4 has been associated with advanced CRC and lymphovascular invasion (26). The incidence of nodal metastasis was significantly higher in CRC patients with CXCR4-positive tumors than in those with CXCR4-negative tumors, and a significant correlation was observed between CXCR4 and vascular endothelial growth factor C expression and lymphatic invasion (27). It was reported that there are more CXCR4-positive cells at metastatic sites in the liver than at primary sites of CRC (28). All brain metastases from CRC were reported to strongly express CXCR4 (29). A high expression of CXCR4 in the primary CRC tumor is considered an independent prognostic factor for poor disease-free survival, and nuclear distribution of CXCR4 is reported to have an inverse relationship with disease-free and overall survival (30). Therefore, CXCR4 is an important mediator of invasion and metastasis of CXCR4-expressing CRC, and it is possible to prevent the development of CRC metastasis through inhibition of CXCR4. The mechanism of growth, invasion, migration and metastasis of CXCL12/CXCR4 pathway includes regulating the expression of MMPs and integrins. CXCR4 and its ligand SDF-1α are inversely expressed in CRC cell lines. SDF-1α activates matrix metalloproteinase-9 and increases vascular endothelial growth factor (VEGF) expression and cell proliferation (31). The concomitant and high expression of CXCR4 and VEGF is a strong and independent predictor of early distant relapse in CRC. CXCR4 triggers a plethora of phenomena, including stimulation of clonogenic growth, induction of VEGF release and ICAM-1 upregulation (32). CXCR4 silencing could upregulate the mRNA and protein expression of E-cadherin, and downregulate the mRNA and protein expression of MMP-2/-9 (33). MMP-2 and MMP-9 expression significantly increased when cells were cultured in the presence of SDF-1α, suggesting that CXCL12-CXCR4 axis triggers increased expression of these genes to promote invasion (34). The CXCR4 ligand SDF-1α doubled secreted VEGFA under hypoxic conditions in human chondrosarcoma cell line and these effects were inhibited by CXCR4 siRNA (35). SDF-1/CXCR4 induces directional migration and liver metastasis of CRC cells by upregulating integrin αvβ6 through ERK/Ets-1 pathway (36). SDF-1 enhanced ovarian cancer cell invasion through αvβ6 integrin-mediated uPA expression via the p38 MAPK and PI3 K/Akt pathway (37). Integrin can modulate tumor cell morphology, and regulate the expression of CXCR4 which is associated with the invasive phenotype and progression of prostate cancer (38).

RNAi is a process in which double-stranded RNA is used to enhance the degradation of cognate mRNA. Synthetic 21–23 nucleotide (siRNA) has been demonstrated to induce transient and efficient RNAi (39). SiRNA-producing plasmid vectors are associated with transient siRNA expression and low transfection efficiency and are widely used in gene interventions, including RNAi (17). In the present study, DNA sequencing and agarose gel electrophoresis provided strong evidence that CXCR4 siRNA was correctly inserted into the multiple cloning site of the GV115 expression plasmid. The physical particle titer of the recombinant virus Lenti-CXCR4-siRNA was 2×10^9^ TU/ml. In the present study, Lenti-CXCR4-siRNA effectively inhibited the expression of CXCR4 mRNA and reduced the level of CXCR4 protein. Lentiviral vector-mediated Met oncogene-specific, stable RNA interference impaired spontaneous motility and invasiveness of canine osteosarcoma cells (40). Lentiviral transgenic systems effectively transferred shRNA targeting human telomerase reverse transcriptase (hTERT) to oral squamous cell carcinoma KB cell lines with >80% gene transfer efficiency, significantly and specifically inhibited hTERT expression both at the mRNA and protein levels, and increased rates of KB cell apoptosis by 206.33% (41). Thus, it is reasonable to conclude that lentivirus can effectively induce gene RNAi.

The increase of tumor cell proliferation and the decrease of apoptosis are essential characteristics of malignant lesions. Our data show that Lenti-CXCR4-siRNA reduces SW480 cell proliferation, which is evidence that the CXCR4/SDF-1 axis stimulates cell multiplication and that inhibition of CXCR4 was able to reduce SW480 cell growth. One study showed that CXCR4 protein expression on the CD34(+) cell surface is lower in the low-grade myelodysplastic syndrome than in high-grade myelodysplastic syndrome; CD34(+) cell apoptosis is significantly higher in low-grade myelodysplastic syndrome, and apoptosis is negatively correlated with CXCR4 expression (42). In another study, CXCR4 RNAi blocked SDF-1-induced proliferation of anaplastic thyroid carcinoma cells and increase in phosphorylation of extracellular signal-regulated kinases (13). A CXCR4 antagonist, d-Arg3FC131, was shown to induce apoptosis and suppress the proliferation of somatotrope tumor cells (15). Thus, CXCR4 RNAi and its antagonist can be used to inhibit tumor cell proliferation and growth.

In the present study, Lenti-CXCR4-siRNA dramatically suppressed the migration and invasion ability of SW480 cells *in vitro*, and reduced the number and mean weight of hepatic metastasis in the liver metastasis model in Balb/c mice. Previously, recombinant CXCR4-RNAi plasmids were found to reduce CXCR4, inhibit cell growth, invasiveness, and migration, and induce cell apoptosis in renal cell carcinoma *in vitro* (7).

Using a recombinant lentiviral RNA interference vector of the CXCR4 gene in highly aggressive (Tca8113 and SCC-9) tumor cells, significant inhibition of the proliferation of both cell lines *in vitro* and *in vivo* was shown (43). Lentivirus-mediated CXCR4 RNAi reduced the expression of CXCR4 and tumor growth, and inhibited metastasis, particularly of bone metastasis of a prostate cancer cell line (44). In a lentiviral CXCR4 overexpression and knockdown model established in SW480, SW620, and RKO cells, CXCR4 overexpression favored chemotaxis and SDF-1α gradient-dependent invasion of cells, while lentiviral-mediated CXCR4 RNAi decreased cell migration (17). Knockdown of CXCR4 with RNAi impaired invasion of breast cancer cells and significantly limited the growth and metastasis to the liver and lung *in vivo* (18,19). CXCL12 stimulation had no impact on Caco-2 cells but significantly increased migration of CXCR4-bearing SW480 and HT-29 cells (CXCR4 expression being less pronounced in HT-29 cells), and this effect was significantly abrogated by neutralizing anti-CXCR4 antibody as well as by CXCR4 siRNAs (25).

In conclusion, hepatic metastasis of CRC is a prevalent and serious problem, and efficacious therapy is required to deal with it. The recombinant lentivirus Lenti-CXCR4-siRNA was correctly constructed in the present study and potently inhibited CXCR4 expression and growth, migration, invasion, and liver metastasis of CRC. Lentiviral mediated CXCR4 RNAi is a potential treatment for CRC.

## Abbreviations:

CXCR4: CXC chemokine receptor 4;
CRC: colorectal cancer;
siRNA: small interfering RNA;
siCXCR4: small interfering CXCR4 RNA
